# The Role of a Good Quality Autopsy in Pediatric Malpractice Claim: A Case Report of an Unexpected Death in an Undiagnosed Thymoma

**DOI:** 10.3389/fped.2020.00031

**Published:** 2020-02-07

**Authors:** Nunzio Di Nunno, Federico Giuseppe Patanè, Francesco Amico, Alessio Asmundo, Cristoforo Pomara

**Affiliations:** ^1^Department of History Society and Human Studies, University of Salento, Lecce, Italy; ^2^Department of Medical, Surgical Sciences and Advanced Technologies G.F. Ingrassia, University of Catania, Catania, Italy; ^3^Departmental section of Legal Medicine “G. Martino”, University of Messina, Messina, Italy

**Keywords:** thymoma, malpractice, autopsy, forensic, liability

## Abstract

Thymomas are extremely rare in the first 20 years of life, with different clinical presentations: from asymptomatic mediastinal masses to compressive and paraneoplastic syndromes. In pediatric population, the respiratory disorders have a higher incidence. The overall thymoma mortality rate is described as 40% and metastasized tumors are more aggressive. This case report describes a compressive syndrome caused by a thymoma in which symptoms were exacerbated by a concurrent pulmonary infection, thus leading an affected infant to sudden death despite medical treatment. In this case, patient's death occurred just before the differential diagnostic process got completed. Malpractice claim was based on the missing diagnosis as well as the suspect of inadequate provided care. Consequently, autopsy played a crucial post-mortem role to find out the cause of death, and to exclude any professional liability. Despite modern diagnostic techniques, autopsies are still the best available forensic tool. It is useful to remember that death is a fact of life, therefore not always preventable.

## Introduction

There are different types of tumors of the thymus, a lymphoid organ situated within the anterior mediastinal compartment, behind the sternum and in front of the great vessels. This organ normally changes over time, reaching its maximum weight at puberty and undergoes involution thereafter. Thymoma is a rare lymphoepithelial mediastinal tumor (1), with only few cases reported, originating from lymphocytes and epithelial cells of the thymus, a lymphoid organ located in the anterior mediastinal compartment. Despite its rareness, this tumor comprises 20–30% of all mediastinal masses in adults, the foremost common primary tumor of the anterior mediastinum. Thymomas are more commonly delineated in adults (70% of cases occur in patients over the age of 40 years) and almost all thymomas occur over the 20th year of age (2). However, thymomas have a broad age distribution (3). Clinical presentation may vary: from asymptomatic mediastinal masses incidentally detected on chest radiographs, to compressive symptoms like dysphagia, venous congestion, or dyspnea. Some of these tumors are related to paraneoplastic syndromes like myasthenia gravis, pure red cell aplasia, immunodeficiency, and connective tissue disorders (4). Symptoms due to compression or direct invasion into adjacent structures may cause a higher incidence of dyspnea or respiratory distress in pediatric patients (5). Approximately 20% of children with thymomas have paraneoplastic syndromes, of which around 70% are myasthenia gravis (6). Because of these aspecific signs, it is imperative that the care provider performs a comprehensive history and physical examination, as the differential diagnosis is extensive and an underlying pathology may be present but underestimated (7). The overall thymoma mortality rate is described as 40% (4). Metastasized thymomas are more aggressive and patients die rapidly due to respiratory failure (8). This case describes a compressive syndrome caused by a thymoma in which symptoms were exacerbated by a concurrent pulmonary infection, thus leading an affected infant to sudden death despite medical treatment.

## Case Report

The forensic pathologist was called by the local prosecutor to investigate the suspicious death of a 4 years-old boy, during hospitalization because of a suspected pulmonary infection.

### Case History

The child was suffering for 15 days from respiratory distress treated by his pediatric doctor with antibiotics and paracetamol. Because of the recent onset of severe respiratory distress, he was admitted to a pediatrics department of a nearby hospital. At admission, he was pale, showing respiratory gasps, reduced vesicular murmur, a blood oxygen saturation of 94% and a heart rate of 161 bpm. A radiograph confirmed the suspect of bronco-pulmonary infection with pleural effusion. Blood examination was consensual (White blood cells count and inflammatory signs were increased). The doctors began therapy with corticosteroids and antibiotics. Considering the not improving situation, the physicians decided to perform a thoracentesis which underwent cytological examination by the pathologists' department, but it was not diagnostic. The hematologic signs, as well as respiratory conditions, were improving only slightly over the following 7 days, then the physicians decided for a CT scan. The CT scan, finally, identified an expansive solid formation in the upper mediastinal compartment, responsive to contrast enhancement. Despite the next diagnostic and treatment efforts, the patient situation worsened unexpectedly, and the child died due to cardiorespiratory failure.

### Autopsy and Histological Findings

**Figure 1:**
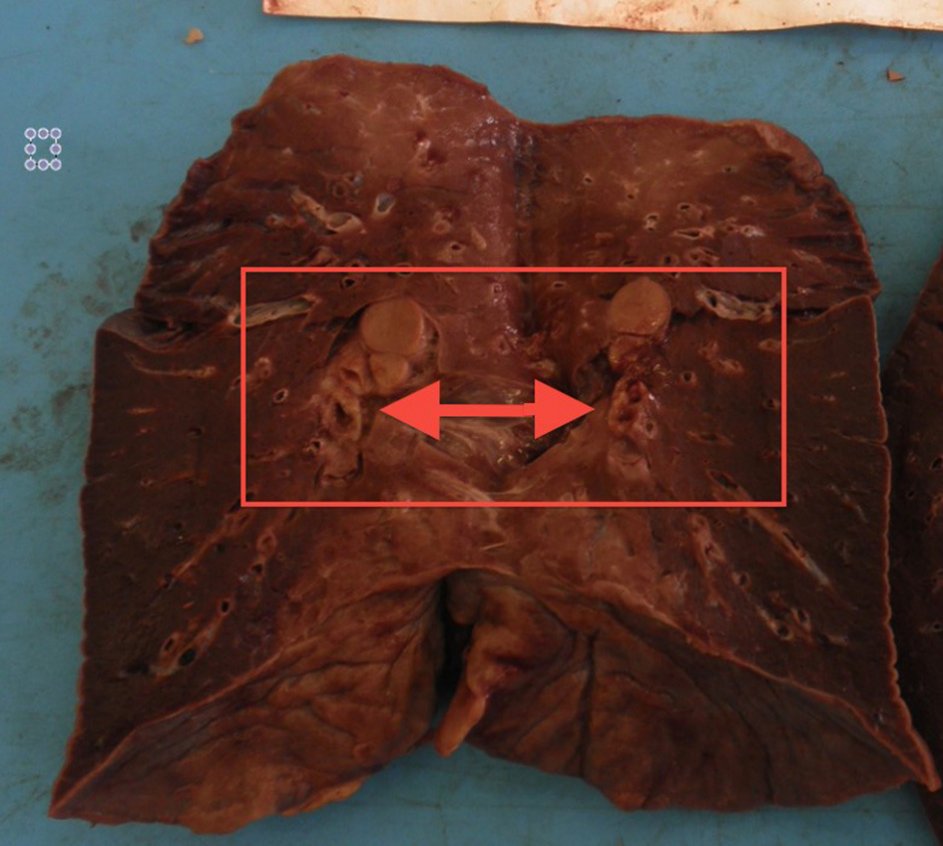
Pulmonary hilum in formaldehyde fixed lung, after vertical section. Gross examination allowed to identify a compression of the vessels and bronchi due to expansive lymph-like masses in the hilum. Similar evidence was found in the other lung.

**Figure 2:**
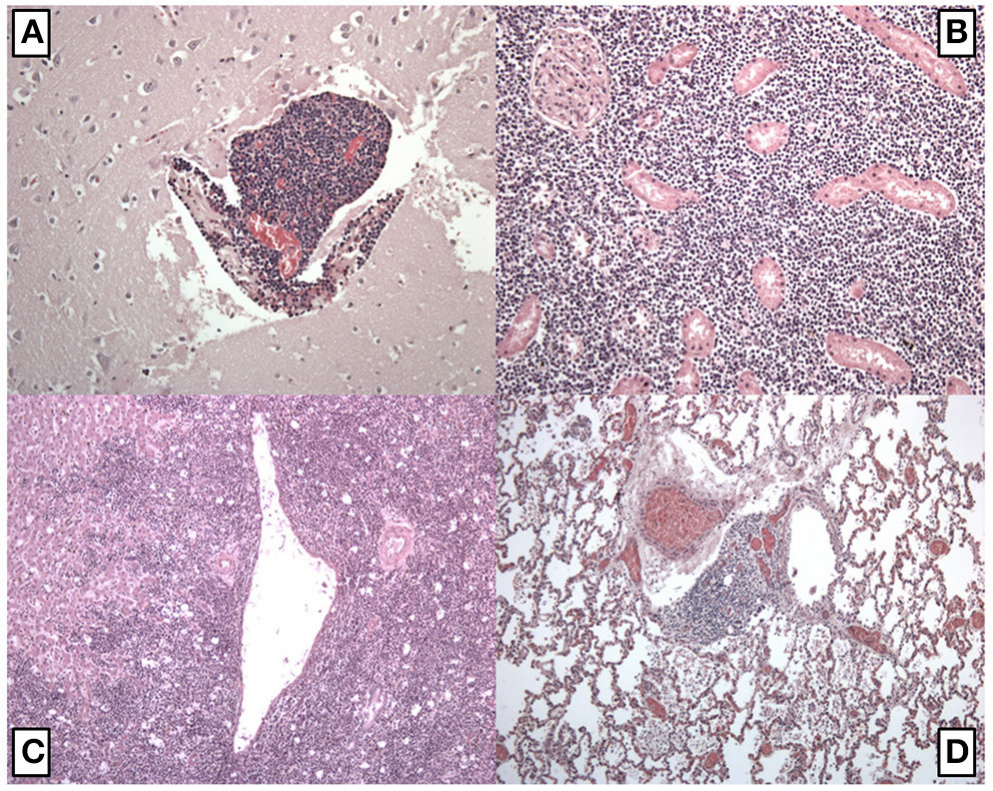
Histological examination after hematoxylin and eosin staining technique. **(A)** Encephalic samples with a lymph-like structure inside the brain tissue; **(B)** kidney samples with an area of lymphocytes invasion; **(C)** liver sample with multiple areas of lymphocytes invasion; **(D)** lung samples with an area of lymphocytes invasion. In some fields was possible to observe granulocytes infiltration areas.

**Figure 3:**
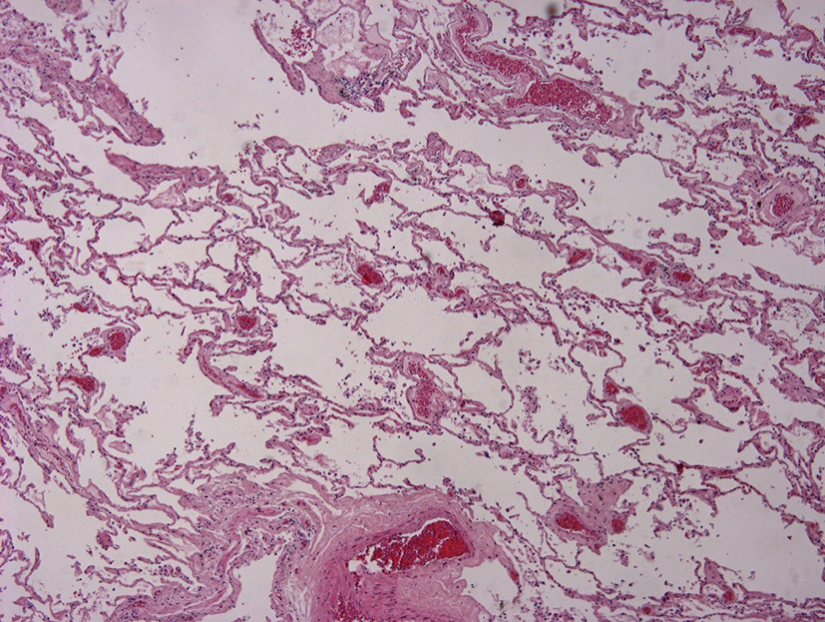
Histological examination after hematoxylin and eosin staining technique. Normal lung samples. Imagine courtesy of Prof. Monica Salerno, Department of Medical, Surgical and Advanced Technologies G.F. Ingrassia, University of Catania.

The autopsy was performed in a layer-by-layer section technique, examining all cephalic, abdominal, and thoracic organs and tissues. Head and neck examination did not show any sign of pathologic process. Thoracic examination was performed after dissecting the chondrocostal margin of the ribs. Several mediastinal adherences were dissected in order to remove the sternum. Inside the mediastinum, an abnormal organ disposition was observed, consisting of a large solid mass composed of two lobes: the right one had a diameter of 10.3 cm while the left one had a diameter of 7.8 cm. This mass was adherent to the trachea, esophagus, aortic arch, and superior pericardial sac, extending longitudinally for 13.7 cm. All specimens were fixed in formaldehyde for further laboratory and histological examinations and stained via hematoxylin and eosin. Some lymph-like masses were found in the hilum of both lungs, causing a compressive displacement of structures (Figure 1). A lymph-like structure surrounded by normal brain tissue was identified in just a single encephalic sample. In almost all cardiac samples it was possible to identify amorphic eosinophilic material occupying interstitial space. In some kidney and liver samples, it was possible to identify similar lymphocyte infiltrations (Figure 2). In some lung samples, it was possible to identify granulocytes invasion areas, whereas in some other samples it was possible to identify lymphocytes. This lung picture is very different if compared with a normal looking lung (Figure 3). The mediastinal mass, microscopically examined after hematoxylin and eosin staining technique was diagnosed as mediastinal neoplasia consisting of elements of small size, with nuclei sometimes convoluted, in high mitotic activity. The presence of numerous macrophages caused a starry sky appearance to the neoplasm with markedly infiltrative character. The morphological aspect was likely of “small cell neoplasms of childhood.” The histological diagnosis was based on the execution of immunohistochemical panels that demonstrated the differentiation line through the detection of antigens: the diagnostic hypotheses included the hematological malignancies deriving from the precursors the lymphoblastic lymphoma that can express phenotype B or T or natural killer; acute myeloid leukemia or myeloblastic sarcoma. Among the morphologically similar non-hematological neoplasms were included some types of sarcomas, such as Ewing's sarcoma and rhabdomyosarcoma, and neural neoplasia derived from the PNET/neuroblastoma type. Immunohistochemical investigations performed after appropriate antigen retrieval showed elements in favor of a lymphoid T neoplasm. The cells expressed an atypical T phenotype with the preservation of CD5 and CD7, partial loss of CD2 and CD3 and lack of CD4 and CDS expression. Neoplastic cells expressed neither CD1a nor CD34 nor Tdt. The latter are markers frequently expressed in lymphoblastic lymphoma. The other differential diagnoses in the context of hematological malignancies were not supported because there was no evidence of expression of Natural killer cytotoxic markers (granzyme, Tia1, perforin) or line B antigens (CD10, CD79a, PAX5) or myeloid lineage (CD68; CD117; MPO). The epithelial marker CAM 5.2 (CK 8/1S), CD99 and synaptofisine were negative. The immunohistochemical findings pointed to a certain diagnosis of a lymphoma deriving from T precursors such as lymphoblastic lymphoma in which the cells reproduce the differentiation stage of cortical thymocyte, “pre-T” due to the absence of CD34 and cD4 and cD8 markers.

### Forensic Results

The cause of death was identified as a cardiorespiratory arrest in a patient affected by a severe mechanical respiratory distress related to severe mediastinal compression by a neoplastic mass, that extended from anterior to superior mediastinal compartment. This mass was a pre-T lymphoblastic lymphoma (cortical thymocytes) which disseminated in several body regions. This compressive action was synergic with the minimal results of a recent post-infective process of the lung. This conclusion is based on the evidences found in the medical documentation, the evidences obtained in autopsy and collected samples examinations, all together with the most significative scientific literature and bibliography. Despite the fact that the physicians did not reach a diagnosis in time to prevent the patient's death, it was not possible to assess any professional liability: no different course of actions could have decreased the chance of death. The death of the young patient was related to the complications of the severe pathologies he was suffering, thus *exitus* was not preventable in any way.

## Discussion

This case shows a death that not respected the WHO criteria of sudden death definition (a non-violent and not otherwise explained, occurring <24 h from the onset of symptoms death) (9), but clinically occurred in an unexpected manner during a slightly improving clinical conditions, just before the conclusion of the diagnostic process. The unexpected death was related to a progressive pathology (lymphoblastic lymphoma) but exacerbated by a concurrent infective process. In fact, while the compressive tumoral mass played a role reducing the adaption of the patient to a distress situation (6), the occurrence of an infective process leaded to a severe respiratory distress. It is useful to highlight that thymomas are rare neoplasms, but the most common tumor of the anterior mediastinal compartment in adults (10). Some researchers have stated that thymomas are extremely rare in patients under 17 years of age (11). Much attention in recent years has been placed on the histological classification of thymoma, since classifications continue to point to clinical staging as the most important parameter for prognosis, particularly for well-differentiated thymic epithelial neoplasms (12, 13). The diagnosis of these tumors has been a topic of controversy for many years and some authors tried to share their experience. According to Suster, clinical features that should raise the possibility of thymoma in the differential diagnosis of a mediastinal mass include: location of the tumor (anterior or anterior-superior mediastinum) described as a circumscribed or lobulated mass surrounded by a fibrous capsule, the absence of malignancy elsewhere via clinical history and patient examination, and concurrent myasthenia gravis or other paraneoplastic syndromes (14). In this case, the patient's death happened just before the differential diagnostic process got completed. The malpractice claim was based on the late diagnosis as well as the suspect of inadequate provided care. Consequently, the autopsy played a crucial post-mortem role to find out the death causes, and to exclude any professional liability. The role of autopsies is to allow doctors to correct, clarify, and confirm ante-mortem clinical diagnosis (15). In this way, physicians may improve their own medical knowledge. Furthermore, they can demonstrate their correct performance and than they can avoid medical malpractice claims. Despite the modern diagnostic techniques, autopsies are still the best available forensic tool (16) and can provide additional information, including findings that if known prior to death may have resulted in improved survival, in more than one third of pediatric deaths (17). Specifically, the autopsy provided results about the pneumonic process while physicians activated all the correct and early protocols. The pneumonic infective process was a concurrent cause of death synergic to the compressive consequences of the thymoma. Epidemiological data of both pathologies were correctly weighted by physicians, who have suspected the most common causes before the rarest, especially among the pediatric population: the pneumonic process has initially caught the physicians' attention, but because of the non-responsiveness to antibiotic treatment, they searched other causes. They eventually suspected of a thymoma due to a CT scan, but unfortunately the clinical conditions worsened too quickly to enact any treatment. Because of the missing specific guidelines, the doctors adopted the best clinical care protocols, so no malpractice claim was demonstrated.

## Conclusions

Rarer are the pathologies and harder is the diagnosis, especially if signs are aspecific. In such cases, if death happens unexpectedly, only the autopsy clarifies the causes of death. Pediatric death review by expert pathologists and clinicians is now widely accepted as an important advance in quality of pediatric care (18). Pediatric thymomas are an example of this kind of pathologies, and if symptoms are too aspecific the only way for a physician to prevent professional liability claims is to adapt to the best healthcare protocols. It is useful to remember that death is a fact of life, therefore not always preventable.

## Ethics Statement

All procedures performed in the study were in accordance with the ethical standards of the institution and with the 1964 Helsinki Declaration and its later amendments or comparable ethical standards. Informed consent was obtained from the relatives.

## Author Contributions

ND, FP, and CP: substantial contributions to the conception or design of the work, or the acquisition, analysis, or interpretation of data for the work. FA and AA: drafting the work or revising it critically for important intellectual content. All authors read and approved the final manuscript.

### Conflict of Interest

The authors declare that the research was conducted in the absence of any commercial or financial relationships that could be construed as a potential conflict of interest.
